# Diphtheria

**Published:** 1876-07

**Authors:** 


### DIPHTHERIA.

At the New York Academy of Medicine, on the evening of March 16th, Dr. C. E. Billington read a paper on this disease and its treatment, which was especially valuable, as it was based entirely upon clinical and personal experience; his observations having been made with great care, and extending over a large number of cases. The records of the Bureau of Vital Statistics showed, said he, that in 1873 there were over four hundred deaths from diphtheria in this city; in 1874 over one thousand, and in 1875 no less than two thousand three hundred and twenty-nine. This terrible epidemic he thought could not be checked by any therapeutic methods, but could only be stamped out by the most revolutionary and active sanitary reform. Dr. Billington has enjoyed unusual facilities for the study of the disease, as he is one of the district physicians of the Demilt Dispensary, and has seen altogether about three hundred cases, of which he has careful records of about one-half.

As a result of his observation and study, he has become fully convinced that diphtheria is a local disease, at least primarily; and, though this is the opinion of a minority of the authorities on the subject, he is glad to have his views corroborated by such observers as Drs. Jacobi and J. Lewis Smith. This conclusion is based upon the following points:

First. The local affection commences first.

Second. The gravity of the general symptoms is in proportion to the severity of the local manifestations.

Third. The results of treatment seem to substantiate this view.

In the study of the nature of the disease, he said, three elements were to be considered:

(1) The *contagium*, which he did not propose to discuss on this occasion.

(2) The inflammation, denuding the fauces of epithelium, and resulting in membranous exudation; and

(3) The effects reflected from the inflammation upon the system in general, are, to a greater or less extent, septicæmic in character.

Dr. Billington's treatment consists mainly in local disinfection, together with the most careful and unremitting watching and attention. The agents which he regards as most useful are the following, in the order in which they stand in his estimation: Tincture of the chloride of iron, lime water and glycerine; and after them, salicylic and carbolic acids, sulphite of sodium, chlorate of potassium, etc. One formula which he uses in almost every case is as follows:

R—Tinct. ferri chlor., f dr. iss.
Glycerinæ.
Aquæ, aa f oz. j.—M.
S.—Teaspoonful every hour or half hour.

Besides being very effective, it has the merit of being pleasant to the taste, which is a great desideratum for children, especially when the dose has to be so frequently repeated. If the child is under two years, one drachm of the tincture of the chloride of iron is enough, and if vomiting follows the administration of the medicine, it should not be given so often.

In connection with the above, Dr. Billington formerly employed the following:

R—Potass. chlor., dr. iss.
Glycerinæ, f oz. ss.
Liq. calcis, f oz. iiss.—M.

A teaspoonful of this was alternated with a dose of the former; so that the patient would receive one or the other every half hour. As a substitute for the chlorate of potassium mixture, he now generally uses the following:

R—Acid salicylic., gr. x—xv.
Sodii sulphit., gr. xxx—xlv.
Glycerinæ, f oz. ss.
Aquæ, f oz. iiss.—M.

Here the salicylic acid is rendered soluble by the addition of three times its weight of sulphite of sodium (borax also has the same effect,) so that in this prescription we have the advantages of both these reputed antiseptics, which are indicated theoretically, and really seem to be of considerable practical benefit. It is of great importance that in every case in which it is practicable some sort of spray should be used upon the throat; and the most convenient instrument with which to accomplish this is the ordinary little perfumery spray-apparatus now in such general use. In order to annoy the child as little as possible, it is best to employ the spray immediately after a dose of the medicine is administered. The combination generally used by Dr. Billington is the following:

R—Acid, carbolic., m.x.
Liq. calcis, f oz. iv.—M.

He believes that the nasal douche or syringe has saved many lives; and even when the nasal passages, apparently, do not seem affected, it is often useful in reaching portions of the mucous membrane inaccessible to the spray. If, therefore, the breath should remain fetid after the employment of the latter, it ought to be resorted to; and the mixture mentioned above, containing the salicylic acid, is as good as any other for the purpose.

In adults or large children it may occasionally be of service to apply carefully strong tincture of iron (say two parts of the tincture to one of glycerine) to circumscribed patches of membrane; but, as a rule, topical applications of caustics or astringents by the probang or camel's-hair brush do much more harm than good, as they cause exhaustion of the little patients from their struggles to resist, excite an increased flow of blood to the part, and really occasion further thickening and spread of the membrane.

Dr. Billington expressed the opinion (which is hardly substantiated by other observers) that quinine is worse than useless in diphtheria in children; being objectionable, if for no other reason, on account of its bitter taste, which makes every dose dreaded by the patient.

In cases attended with high secondary fever, a full dose of quinine, worked better in his hands. He cannot subscribe to the prevalent opinion that diphtheria will never bear antiphlogistic treatment.

Dr. Billington then proceeded to give an interesting summary of the cases which he had personally observed, prefacing his statement with an allusion to the well recognized disadvantages to be encountered in dispensary practice. According to his observations, about sixty-five per cent. of all cases of diphtheria occur in persons under five years of age, and it is quite a rare affection among adults (except in the peculiar experience of certain irregular practitioners,) even when individuals are constantly and to the fullest extent exposed to the disease. He has also found that about sixty per cent. of all the cases will recover without any treatment at all, and that about five per cent. will prove fatal whatever plan may be adopted. Out of one hundred and two carefully tabulated dispensary cases treated by him, fourteen died, and eighty-eight recovered; while of seventeen cases in private practice, one died, and sixteen recovered.

The usual duration of the attack, from the commencement of the treatment to the disappearance of the diphtheritic membranes, was only from four to six days. Twenty-four cases in private practice, treated on the same principles by Dr. William Darken, house physician to the Demilt Dispensary, show even a better result; as not a single death occurred directly from the disease, though one of the children died several weeks after the acute attack, from some unexplained cause.

A still later series of fourteen cases treated by Dr. Billington in conjunction with Dr. W. E. Bullard (in order that the patients might receive the fullest possible amount of attention) all recovered, so that we have fifty-five cases altogether, with only one death directly attributed to the disease. In a large number of these the attack was of very great severity.

From his observations, Dr. Billington has been induced to believe that a laryngeal complication can often be prevented or aborted by the use of the spray, and that even after the membranes have been fully formed in this locality it is of very great service. Calomel has also proved useful in many cases. The inhalation of *hot* vapor, he thinks, renders the system more favorable to the absorption of septic materials, and therefore injurious.

He did not express a positive opinion as to the identity of croup and diphtheria, but apparently seemed to hold to the former view.—*Canada Lancet.*

